# Temporal properties of positive and negative defocus on emmetropization

**DOI:** 10.1038/s41598-022-07621-6

**Published:** 2022-03-04

**Authors:** Xiaoying Zhu, Pauline Kang, David Troilo, Alexandra Benavente-Perez

**Affiliations:** 1 College of Optometry, State University of New York, 33 West 42nd Street, New York, NY 10036 USA; 2 School of Optometry and Vision Science, University of New South Wales, Sydney, NSW 2052 Australia

**Keywords:** Visual system, Visual system

## Abstract

Studying the temporal integration of visual signals is crucial to understand how time spent on different visual tasks can affect emmetropization and refractive error development. In this study we assessed the effect of interrupting positive and negative lens-imposed defocus with brief periods of unrestricted vision or darkness. A total of forty-six marmosets were treated monocularly with soft contact lenses for 4 weeks from 10 weeks of age (OD: + 5D or − 5D; OS: plano). Two control groups wore + 5D (n = 5) or − 5D (n = 13) lenses continuously for 9 h/day. Two experimental groups had lens-wear interrupted for 30 min twice/day at noon and mid-afternoon by removing lenses and monitoring vision while marmosets sat at the center of a viewing cylinder (normal vision interruption, + 5D: n = 7; − 5D: n = 8) or while they were in the dark (dark interruption, + 5D: n = 7; − 5D: n = 6). The interruption period (30 min/day) represented approx. 10% of the total stimulation time (9 h/day). On-axis refractive error (RE) and vitreous chamber depth (VCD) were measured using an autorefractor and high frequency A-scan ultrasound at baseline and after treatment. Wearing + 5D lenses continuously 9 h/day for 4 weeks induced slowed eye growth and hyperopic shifts in RE in treated relative to contralateral control eyes (relative change, VCD: − 25 ± 11 μm, p > 0.05; RE: + 1.24 ± 0.58 D, p > 0.05), whereas − 5D lens wear resulted in larger and myopic eyes (relative change, VCD: + 109 ± 24 μm, p < 0.001; RE: − 2.03 ± 0.56 D, p < 0.05), significantly different from those in the + 5D lens-treated animals (p < 0.01 for both). Interrupting lens induced defocus with periods of normal vision or darkness for approx. 10% of the treatment time affected the resulting compensation differently for myopic and hyperopic defocus. Interrupting defocus with unrestricted vision reduced − 5D defocus compensation but enhanced + 5D defocus compensation (− 5D, VCD: + 18 ± 33 μm; RE: − 0.93 ± 0.50 D, both p > 0.05; + 5D, VCD: − 86 ± 30 μm; RE: + 1.93 ± 0.50 D, both p < 0.05). Interrupting defocus with darkness also decreased − 5D defocus compensation, but had little effect on + 5D defocus compensation (− 5D, VCD: + 73 ± 34 μm, RE: − 1.13 ± 0.77 D, p > 0.05 for both; + 5D, VCD: − 10 ± 28 μm, RE: + 1.22 ± 0.50 D, p > 0.05 for both). These findings in a non-human primate model of emmetropization are similar to those described in other species and confirm a non-linear model of visual signal integration over time. This suggests a mechanism that is conserved across species and may have clinical implications for myopia management in school-aged children.

## Introduction

Emmetropization is a highly coordinated developmental process of postnatal eye growth. The role that vision plays in emmetropization has been confirmed across multiple species including mice^1,2^, chicks^3,4^, guinea pigs^5,6^, treeshrews^7–9^, marmosets^10,11^ and rhesus monkeys^12,13^. The spatial and temporal characteristics of the visual experience have an impact on the emmetropization process^14,15^. When viewing the three-dimensional world, the eye experiences different types and magnitudes of defocus across the retina (spatial integration), which vary rapidly over time (temporal integration). The eye integrates this dynamic combination of visual signals both temporally and spatially across the retina to guide its growth^14,16^. Gaining a better understanding of the spatial and temporal integration of visual signals involved in emmetropization will contribute towards our understanding of refractive error development, and the advancement of more effective approaches for myopia management in children.

Visual signals also actively modify eye growth in young animals bidirectionally. Depriving the eye of form vision typically results in axial myopia, a phenomenon termed form deprivation myopia^10,17–19^. The response is dose dependent; the greater the visual degradation the greater the induced axial myopia^20–23^. Recovery from form deprivation by providing normal vision^3,24^ and more importantly, lack of recovery when the myopia induced by form deprivation was corrected with spectacle lenses^25,26^, provide further evidence that eye growth is regulated by visual signals. Changes to the retinal image through defocus also stimulate compensatory growth responses across a variety of species. Myopic defocus induced by positive lenses slows eye growth and thickens the choroid, which moves the retina forward to meet the new focal plane. The opposite occurs in response to hyperopic defocus. However, the eye’s response to myopic and hyperopic defocus is not symmetric and visual signals generated in response to myopic retinal defocus appear to be more potent than those of hyperopic defocus^27–31^.

In terms of the temporal integration of visual signals, studies have demonstrated that ocular growth and refractive compensatory responses to defocus are significantly affected by the temporal characteristics of the stimulus. The frequency and duration of exposure, rather than the total amount of defocus exposure, have a greater influence on eye growth, supporting a non-linear model of temporal integration of visual signals^16^. Chick eyes exposed to frequent, repeated episodes of lens wear developed greater lens compensation than less frequent episodes of the same total duration^32,33^. Furthermore, as little as six episodes of 2-min exposure to darkness prevented the development of experimentally induced myopia from day-long negative lens wear^31^. When positive and negative lenses are worn alternately, visual signals generated in response to myopic defocus dominate and dictate eye growth, confirming that visual signals are not simply averaged over time^29,31^.

A recent study from our laboratory described the impact of interrupting negative daily lens-wear with two 30-min periods of unrestricted vision in a viewing cylinder^34^. The brief interruptions reduced the eyes’ compensatory response, resulting in less myopia and smaller eyes. The interruptions had a greater impact in reducing the compensatory response if it occurred during myopia development, than if eyes had already developed myopia^34^. Interrupting hyperopic defocus has also shown to prevent or decrease myopia development in chickens^35^, guinea pigs^36^, tree shrews^37^, and rhesus monkeys^30^. Brief periods of clear vision can also diminish axial elongation induced by hyperopic defocus in human eyes^38^.

Gaining insights into the temporal dynamics of visual information integration is essential to understand emmetropization and refractive error development. In the current study, we evaluated the impact of interrupting myopic or hyperopic retinal defocus on the eye’s compensatory response with short periods of normal vision compared to periods of darkness.

## Methods

### Experimental protocol

**Figure 1:**
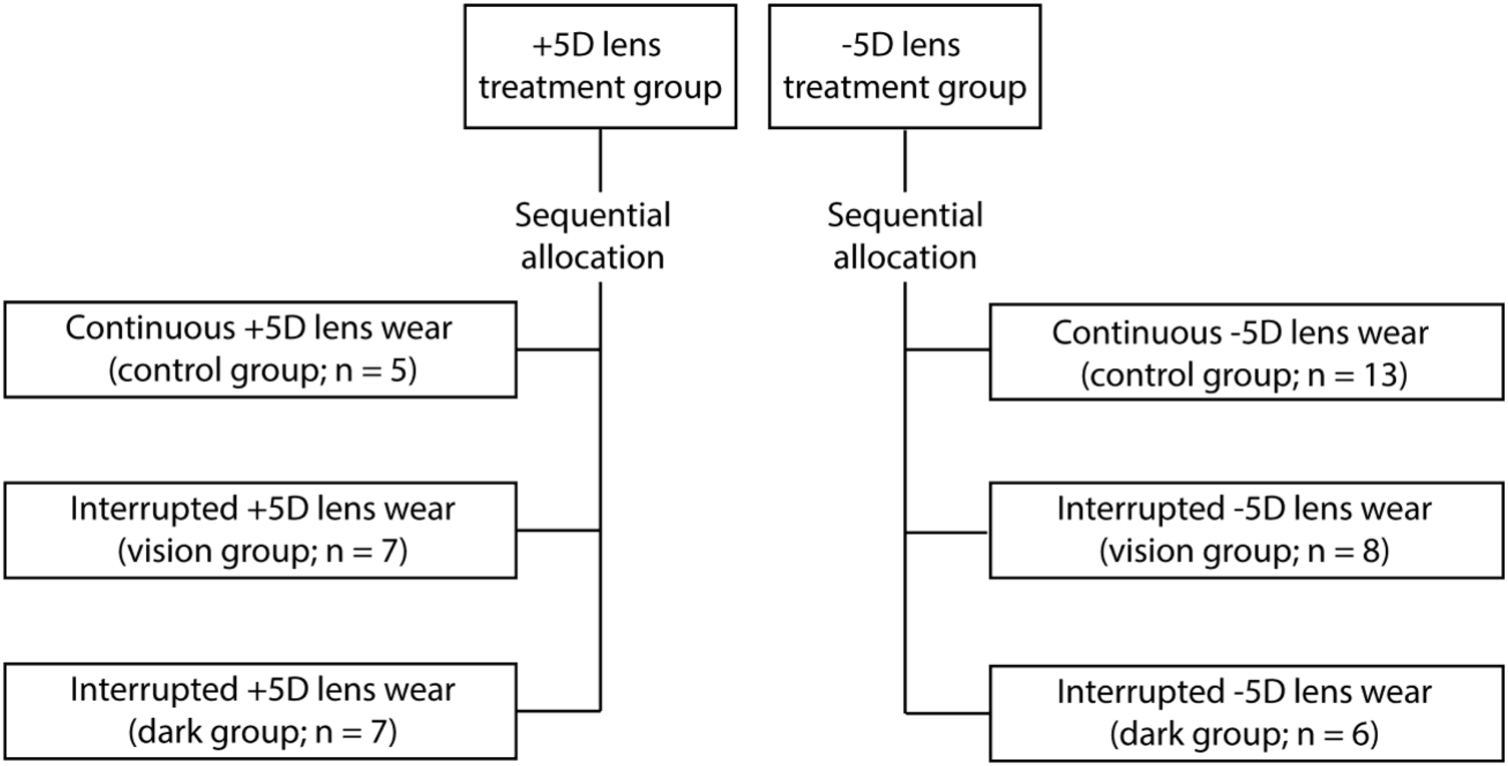
Sequential allocation of marmosets in the control and treatment groups for both + 5D and − 5D lens wear. Note that the sample size of the − 5D lens wear control group is larger compared to other groups as it included historical data.

A total of 46 juvenile marmosets (*Callithrix jacchus*) were randomly treated with either + 5D or − 5D soft contact lenses. Artificial lighting was provided using daylight-balanced fluorescent lamps on a 9:15 light:dark diurnal cycle (700 lux). Lenses were inserted at light onset (700 lux) at around 9 am (± 1 h) and removed at lights off each day (9 h light/15 h dark). Within each treatment arm, marmosets were assigned to the control group (continuous lens wear), or one of two treatment groups with interrupted lens wear for 30 min, twice a day (12 pm and 3 pm), during which lens wear was interrupted with unrestricted vision (vision group) or darkness (dark group). During lens wear interruption, marmosets were placed inside a viewing cylinder under normal light so they had unrestricted vision or kept in their housing environment in the dark (Fig. 1). The drum was built to allow normal vision at a controlled distance while the animal’s dynamic refractive state was monitored. All animal care, treatment and experimental protocols were reviewed and approved by the State University of New York College of Optometry Institutional Animal Care and Use Committee and conformed to the ARVO Statement for the Use of Animals in Ophthalmic and Vision Research.

All animals were treated with a single vision positive (+ 5D) or negative (− 5D) soft contact lens on their right eye (experimental eye, X), and a plano lens on the fellow eye (control eye, N). Contact lenses had base curves of 3.6 mm or 3.8 mm, and total diameters of 6.0 mm or 6.5 mm. Lens were made from methafilcon A (55% water content, DK: 17) and fitted 0.10 mm flatter than the flattest keratometry measurement.

Lens treatment began at approximately 10 weeks of age (71.20 ± 1.04 days) for a duration of four weeks. No corneal complications related to contact lens wear were observed in any of the animals treated in this or earlier studies with marmosets^34,39,40^.

**Figure 2:**
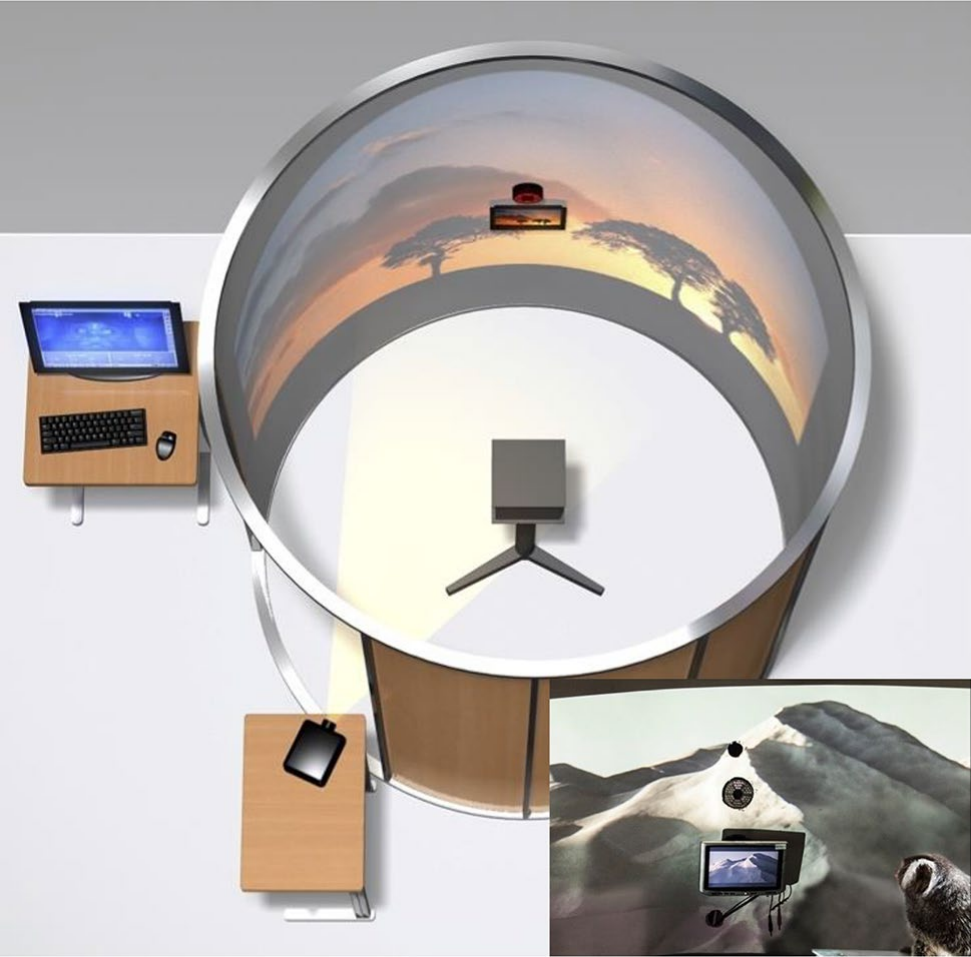
The viewing cylinder the marmosets were in during lens interruption. The inset is a photo of the natural scenes projected on the walls of the viewing cylinder (photographed and rendered by William Bourassa). Marmosets viewed these natural scenes when lens wear was interrupted with unrestricted vision.

Marmosets in the control group wore + 5D or − 5D lenses continuously during all light hours. The normal vision interruption protocol consisted of wearing lenses for 3 h in the morning and removing them for 30 min at 12 pm (± 1 h) during which the marmosets were kept inside a viewing cylinder with a 1-m radius (Fig. 2). Marmosets were gently restrained in a primate chair and viewed natural scenes projected on the walls of the viewing cylinder (Fig. 2)^34^. The defocus that marmoset eyes experienced was monitored during the unrestricted vision interruption (when the contact lenses were removed) using an infrared video photorefractor (PowerRefractor, MultiChannel Systems, Tubingen, Germany). Lenses were then reinserted and worn for the following 3 h, removed for 30 min around 3 pm (± 1 h), during which marmosets were exposed to the same environment. Lenses were reinserted again for 3 additional hours before removal for the night at approximately 6 pm (± 1 h). For the dark interruption protocol, their housing environment was covered for 30 min with a light-proof cover at 12 pm and 3 pm. The light-proof cover did not alter the housing temperature or animal behavior.

This procedure was repeated every day during the 4 weeks of treatment.

### Outcome measures

Study measurements were taken at baseline and after 4 weeks of lens treatment 20 min after cycloplegia (2 drops of 1% cyclopentolate).

Ocular biometry including anterior chamber depth, lens thickness, vitreous chamber depth (VCD), retinal thickness, choroidal thickness and axial length was measured using a high frequency A-scan ultrasound (25 MHz, Panametrics, NDT, Ltd, Waltham, MA). Cycloplegic axial refraction (RE) was measured using the Nidek ARK-900 autorefractor (Gamagori, Japan) and an average of 5 measurements was reported^34,39,40^. All the procedures were performed in accordance with ARRIVE guidelines.

### Statistical analysis

Data are shown as mean ± standard error of the mean (SEM). To compare the effect of continuous vs interrupted lens treatments, we used the relative change from baseline in the interocular difference (the change in the lens-treated eye over the course of the experiment minus the change in the fellow eye), to account for the change in the fellow, untreated eyes. To compare the effect of interrupting positive vs negative lens wear, the percentage relative change (the ratio between the relative change in the interruption group and the control group) was calculated, and unpaired two-tailed Student’s t-tests were used to compare normal vision vs dark interruption conditions. Microsoft Excel (Version 16.56, WA) was used for these analyses. Lastly, Analysis of Variance (ANOVA) was used to compare the relative change in the three groups for each type of lens wear using DataDesk (Version 8.1.0, NY).

## Results

Below we describe the effect that treatment had on RE and VCD. A summary of the raw data for all the biometric parameters and RE is included in Supplementary Tables S1 (positive lens wear) and Supplementary Table S2 (negative lens wear).

### Effect of continued lens treatment

**Figure 3:**
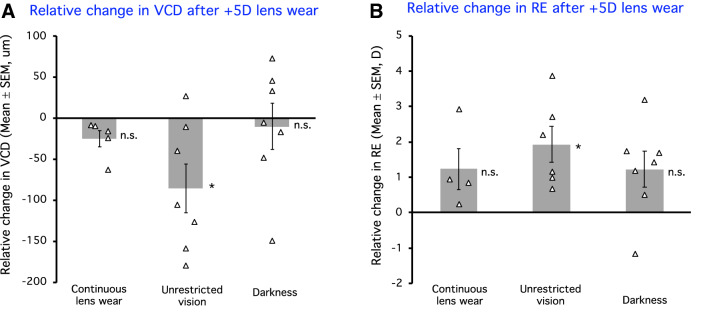
The relative change (the change in the lens-wearing eye relative to the change in the fellow eye) in vitreous chamber depth (VCD, **A**) and refractive error (RE, **B**) after wearing + 5D lenses for 4 weeks, either continuously or with interruptions. Triangles show individual animals, and bars represent the average for each group. RE was measured in 4 out of the 5 animals for the control group, and in 6 out of 7 animals for the group with unrestricted vision. *n.s.* Not significant; *p < 0.05.

Animals treated continuously with + 5D lenses without interruptions developed a decrease in vitreous chamber depth and a hyperopic shift in the treated eyes relative to contralateral control eyes (mean relative change, VCD: − 25 ± 11 μm, Fig. 3A; RE: + 1.24 ± 0.58D, Fig. 3B; p > 0.05 for both).

**Figure 4:**
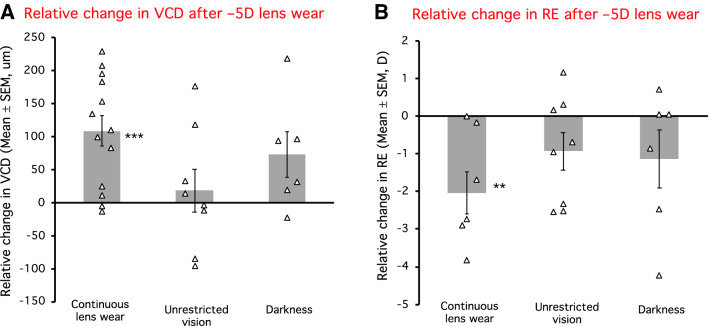
The relative change (the change in the lens-wearing eye relative to the change in the fellow eye) in vitreous chamber depth (VCD, **A**) and refractive error (RE, **B**) after wearing − 5D lenses for 4 weeks, either continuously or with interruptions. Triangles show individual animals, and bars represent the average for each group. RE was measured in 6 out of the 13 animals for the control group. *n.s.* Not significant; **p < 0.01; ***p < 0.001.

Wearing − 5D lenses continuously for 4 weeks caused an increased vitreous chamber elongation and a myopic shift in treated relative to fellow eyes (mean relative change, VCD: + 109 ± 24 μm, p < 0.001, Fig. 4A; RE: − 2.03 ± 0.56D, p < 0.01, Fig. 4B).

The compensatory response in eye growth and refractive state to imposed defocus with − 5D or + 5D lenses differed significantly (p < 0.01 for VCD and RE).

### Effects of interrupting defocus with unrestricted vision

**Figure 5:**
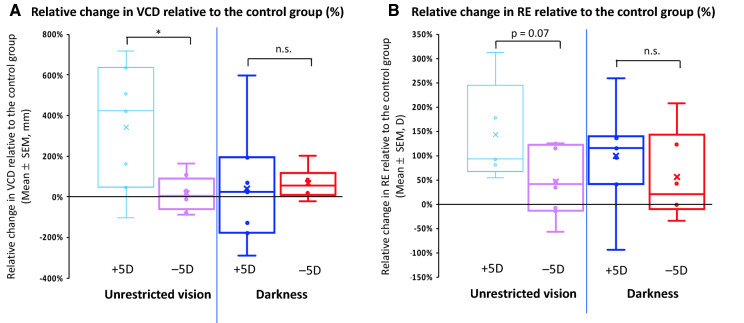
The percentage change between the groups with interrupted lens wear and the control group (the ratio between relative change in an interruption group and that in the control group) for vitreous chamber depth (VCD, **A**) and refractive error (RE, **B**). *n.s.* Not significant; *p < 0.05.

A striking finding in this study is that interrupting + 5D imposed defocus with unrestricted vision twice/day enhanced compensation in vitreous chamber depth resulting in greater hyperopia compared with continuous lens wear (mean relative change, VCD: − 86 ± 31 μm, Fig. 3A; RE: + 1.93 ± 0.50D, Fig. 3B, p < 0.05 for both). This is 241% more VCD compensation and 43% more hyperopia compared to the control group with + 5D lens wear (Fig. 5A,B).

Interrupting − 5D lens wear with normal vision, on the other hand, reduced negative lens compensation and resulted in slower eye growth and a smaller myopic shift (mean relative change, VCD: + 18 ± 33 μm, Fig. 4A; RE: − 0.93 ± 0.50D, Fig. 4B; p > 0.05 for both), which represented 18% and 46% of the total VCD and RE compensation to continuous − 5D lens wear, respectively (Fig. 5A,B).

The difference in compensatory responses measured as the percentage change of interrupting + 5D compared to − 5D lens wear reached significance for the vitreous chamber compensation (341% vs. 18%, p < 0.05; Fig. 5A) and approached significance for the refractive compensation (143% vs. 46%, p = 0.07, Fig. 5B).

**Figure 6:**
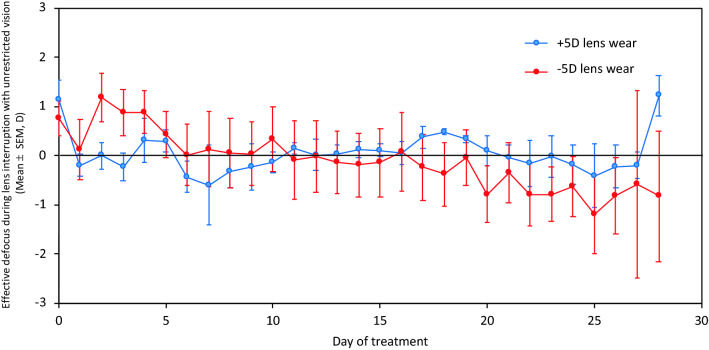
Daily average of the defocus experienced by marmosets during the normal light interruption while they were kept inside a cylinder on which natural scenes were projected. The defocus in the + 5D group is represented in blue, and the defocus in the − 5D group is shown in red.

The defocus that marmoset eyes experienced was monitored during the unrestricted vision interruption (when the contact lenses were removed). The wall of the viewing cylinder was 1 m away from the animal, which superimposed + 1D of hyperopic defocus in an emmetropic, unaccommodating eye. The average effective defocus throughout the 4 weeks of treatment was hyperopia for the group treated with + 5D lenses (+ 0.05 ± 0.08 D). For the group treated with − 5D lenses, there seemed to be a hyperopic shift (+ 1.15 ± 0.47 D) earlier in the treatment, perhaps caused by relaxed accommodation. The treated eyes in this group experienced approximately 1 D of myopic defocus at the end of the treatment. The average effective defocus for this group was − 0.10 ± 0.11 D (Fig. 6).

### Effects of interrupting imposed defocus without vision (darkness)

Interrupting + 5D lens wear with darkness twice/day reduced the inhibitory effect of positive lenses on VCD but had little effect on the compensatory hyperopic shift (mean relative change, VCD: − 10 ± 28 μm, Fig. 3A; RE: + 1.22 ± 0.54 D, Fig. 3B; p > 0.05 for both). These interrupted compensations represent 41% and 99% of VCD and RE compensation observed in the continuous + 5D group, respectively (Fig. 5A,B).

In contrast, interrupting − 5D lens wear with darkness reduced vitreous elongation and negative lens refractive compensation (mean relative change, VCD: + 73 ± 34 μm, Fig. 4A; RE: − 1.13 ± 0.84 D, Fig. 4B; p > 0.05 for both). The resulting compensation represented 67% and 56% of the VCD and RE compensation observed in the − 5D continuous group, respectively (Fig. 5A,B).

The difference in percentage changes of interrupting + 5D and − 5D lens wear was not significant for either VCD or RE (VCD: 41% vs. 67%, Fig. 5A; RE: 99% vs. 56%, Fig. 5B; p > 0.05 for both).

### Predictors of compensatory changes in VCD

Multiple regression analyses showed that thickness of the crystalline lens, retina, and choroid in the experimental eyes can predict the change in VCD in certain situations. In eyes whose + 5D lens wear was interrupted with darkness, eyes that grew less at the end of lens treatment had thinner lens and thicker choroids at baseline (both p < 0.05, R^2^ = 0.76). For the group with continuous − 5D lens wear, eyes that exhibited more vitreous chamber elongation at the end of lens treatment had thicker lens (p < 0.01) and retina (p < 0.05) at baseline (R^2^ = 0.76).

## Discussion

Interrupting experimentally imposed defocus from + 5 or − 5 D contact lens wear with brief periods of unrestricted vision or darkness resulted in changes in the degree of compensation. Our results support the idea that the integration of the defocus signals for emmetropization is non-linear in nature. Interrupting monocular negative lens treatment for approximately 10% of the total lens-wearing time with unrestricted vision reduced compensatory myopia by more than 50%, but interrupting monocular positive lens treatment by the same amount did not reduce the development of compensatory hyperopia. Interestingly, interrupting monocular positive lens wear with unrestricted vision actually enhanced the compensation for the lens-imposed defocus. Note that binocular negative lens treatment has been shown to induce a higher degree of compensation in marmosets^41^.

It has been proposed from earlier work that the temporal integration of visual signals exhibits three non-linearities^16^: (1) The retina does not weight myopic and hyperopic defocus equally: Short periods of normal vision reduce experimental myopia caused by wearing negative lenses but have minimal effects on experimental hyperopia caused by wearing positive lenses; (2) lens compensation depends on the frequency and duration of individual episodes of lens wear, not just the total exposure of lens wear per day; and (3) lens compensation strongly depends on the sign of defocus and the initial state of ocular components (particularly axial length and choroidal thickness).

Our results show that interrupting negative lens wear with normal vision or darkness for 30 min twice a day (approx. 10% of their total daily exposure) reduced negative lens compensation by approximately 50%, confirming the transient nature of the compensatory growth response to negative defocus when the eye has not yet developed myopia^35^. In addition, our results further support the notion that lens compensation does not depend on the total exposure of lens wear per day. Rhesus monkey eyes that had four daily 15-min episodes of unrestricted vison eliminated the refractive compensation to 11 h of − 3D lens wear^30^; whereas marmoset eyes with two daily 30-min episodes of normal vision reduced refractive compensation to 9 h of − 5D lens wear by 54%. The effectiveness of the interruption in reducing the compensation for imposed defocus sheds light on the strength of the emmetropization signals. We hypothesize that the strength of the signals may vary as function of the degree of eye growth. Interruptions of the signals may have greater effects during the early phase of compensation. Once compensatory growth is underway or nearly complete it may require longer interruptions to be effective.

The temporal integration of the visual signals used for emmetropization also differs for myopic and hyperopic defocus. The temporal features of the emmetropization signal regulating axial compensation for defocus has been confirmed in chicks^16^. The rise and saturation of the signal during lens wear has similar temporal patterns for both positive and negative lens wear, but the decay of the signal between lens wear exhibits distinctively different temporal patterns: The signal for positive lens compensation decays much slower compared with the signal for negative lens compensation^28^. As such, the effect of positive defocus on axial length is longer lasting and more robust than the effect of negative defocus. For instance, interrupting positive lens wear with one daily 3-h period of normal vision (25% of total lens wear) reduced positive lens compensation only by 8% in chicks, whereas interrupting negative lens wear by the same duration eliminated negative lens compensation^35^. Our results in marmosets show that interrupting + 5D lens wear with unrestricted vision actually increased positive lens compensation, suggesting that the intrinsic emmetropization signal does not decay between episodes. In fact, this signal may rise even faster with interruptions providing unrestricted vision, suggesting that the weighting of myopic defocus signals can be influenced by temporal changes in visual experience. In contrast, our results show that interrupting − 5D lens wear by unrestricted vision or darkness for a total of one hour (11% of total lens exposure) reduced compensatory myopia by approximately 50%, suggesting that hyperopic defocus signals may decay rapidly between interruptions.

Why interrupting positive lens wear caused more compensation is unknown. It is possible that the eye growth control characteristics was altered by the repeated lens interruption with vision. Lens compensation to imposed retinal defocus is a closed-loop feedback system where eye growth is controlled by visual input. Eyes appear to compensate for retinal defocus until it is reduced to a set-point^14^. Furthermore, optical correction prevented eyes from recovering from induced myopia in tree shrews^42^ and chicks^25^. Monitoring the defocus during positive lens interruptions revealed that eyes on average experienced very little defocus (0.05D). Alternating between 5D of myopic defocus and emmetropia may have changed the eye growth controller’s response characteristics. It is possible that interrupting positive lens wear repeatedly with vision somehow altered the threshold of the eye growth controller, resulting in an exaggerated response to myopic defocus. Alternatively, it is possible that interrupting positive lens wear with normal vision increased the system’s gain so the response to the defocus signal rose faster between episodes. That the enhanced response was seen only for interruption of imposed positive defocus seems to be related to the different responses to positive and negative defocus. This is supported by the hypothesis that defocus of opposite signs affect eye growth via largely distinct retinal pathways^43^.

**Figure 7:**
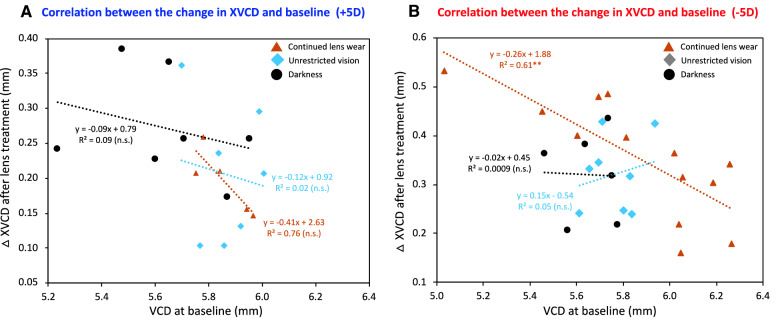
Changes in vitreous chamber depth (VCD) as a function of the baseline measurement for animals wearing + 5D (**A**) and − 5D (**B**) lenses. *n.s.* Not significant; **p < 0.01.

The growth of the eye and its refractive state may affect the temporal integration of lens compensation. We found negative correlations between the change in the depth of the vitreous chamber of the experimental eyes over the course of the experiment as a function of their vitreous chamber depth at baseline (Fig. 7). The larger eyes at the baseline tended to elongate less during the experiment, whereas shorter eyes at the baseline tended to elongate more, regardless of lens treatment. This trend was disrupted by lens interruption, shown by the reduced slopes in linear regressions found in the animals whose lens wear was interrupted by unrestricted vision or darkness. However, the comparisons of slopes between the control groups and the interrupted groups did not reach statistical significance.

While it is well established that visual input plays an important role in the modulation of eye grow and refractive state, there may also be some intrinsic, non-visual factors regulating eye growth. For example, chick eyes that had elongated excessively and developed hyperopia because of consistent dark rearing continued to elongate more than normal, partially offsetting the hyperopia despite the lack of visual input^44^. A recent study showed that chick eyes can recover from prior positive and negative lens wear while kept in the darkness (to eliminate any visual input)^45^. Furthermore, it was speculated that there may be an endogenous, possibly genetic, definition of the set-point of emmetropization in each chick^46^. Perhaps a similar phenomenon also exists in marmoset eyes.

Our results suggest that interrupting negative lens wear with unrestricted vision may help slow myopia progression and axial elongation in school-aged children. It has been speculated that correction of axial myopia may increase peripheral hyperopia, particularly in prolate-shaped eyes, possibly leading to increased myopia progression^47^. Measurement of power profiles of single-vision soft contact lenses show that some commercially available contact lenses have a negative spherical aberration profile that would exacerbate myopia progression^48^. Our findings that interrupting hyperopic defocus imposed by negative lens wear with unrestricted vision for 11% of the time of visual experience reduced myopia development by more than 50% suggest that periodically removing the negatively powered spectacles while the eyes are exposed to a normal environment may help reduce myopia progression in children. Swiatczak and Schaeffel recently suggested that the retina of a myopic eye has reduced ability to detect positive defocus as myopic eyes in young adults elongated similarly after watching movies that were either filtered (with calculated defocus) or with real positive defocus for 60 min^49^. While this possibility may exist, it is worth noting that this experiment was conducted on young adults (average age 24 ± 4 years), not school-aged children, and that subjects were exposed to the defocus for only 60 min. These findings do not necessarily suggest that myopic children may not respond to positive defocus. In fact, numerous clinical trials have demonstrated that myopic children do respond to myopic defocus and therefore, myopic defocus is widely used, by wearing multifocal contact lenses, orthokeratology, progressive addition lenses or bifocal glasses, to slow myopia progression in children^50^. More research is needed to further elucidate the mechanism(s) involved in emmetropization and integration of visual signals, to maximize the efficacy of myopic defocus on myopia management in children.

## Conclusion

We report in a non-human primate model of emmetropization that visual signals are temporally integrated in a non-linear fashion: (1) Interrupting positive and negative lens wear with brief periods of unrestricted vision had differential effects on lens compensation: while interrupting negative lens wear decreased axial growth and the development of myopia, interrupting positive lens wear enhanced the development of axial hyperopia. (2) Although not statistically significant, interrupting lens wear with brief periods of darkness tended to reduce negative lens compensation more than positive lens compensation. Our results suggest that removing single-vision myopia corrections for brief periods daily during waking hours may help slow myopia progression in school-aged children.

## Supplementary Information

Supplementary Table S1.

Supplementary Table S2.
