# Supplementary material for: Temporal properties of positive and negative defocus on emmetropization
Source: Sci Rep. 2022 Mar 4;12:3582. doi: 10.1038/s41598-022-07621-6 (PMC8897502; doi:10.1038/s41598-022-07621-6)
Supplement: Supplementary file 1 — Supplementary Table S1. [file 41598_2022_7621_MOESM1_ESM.pdf]

Supplemental Table S1. Ocular biometry and refractive state for the experimental (X, OD) and control eyes (N, OS) at baseline and after 4 weeks of +5D lens treatment. Data are shown as mean ± SEM.

	Treatment	Baseline		4 weeks later		p (t-test)	p (ANOVA)
		X (OD)	N (OS)	X (OD)	N (OS)		
**Anterior chamber depth (mm)**	**Control (n = 5)**	1.55 ± 0.02	1.53 ± 0.02	1.60 ± 0.01	1.59 ± 0.02	> 0.05	> 0.05
	**Unresticted vision (n = 7)**	1.52 ± 0.02	1.52 ± 0.02	1.59 ± 0.02	1.59 ± 0.02	> 0.05	
	**Darkness (n = 7)**	1.51 ± 0.02	1.51 ± 0.02	1.59 ± 0.02	1.58 ± 0.01	> 0.05	
**Lens thickness (mm)**	**Control (n = 5)**	2.05 ± 0.03	2.04 ± 0.02	2.01 ± 0.01	2.02 ± 0.02	> 0.05	> 0.05
	**Unresticted vision (n = 7)**	2.06 ± 0.02	2.06 ± 0.02	2.05 ± 0.03	2.05 ± 0.02	> 0.05	
	**Darkness (n = 7)**	2.09 ± 0.02	2.08 ± 0.02	2.03 ± 0.01	2.07 ± 0.03	> 0.05	
**Vitreous chamber depth (mm)**	**Control (n = 5)**	5.86 ± 0.04	5.88 ± 0.05	6.05 ± 0.03	6.10 ± 0.04	> 0.05	> 0.05
	**Unresticted vision (n = 7)**	5.87 ± 0.04	5.88 ± 0.04	6.07 ± 0.05	6.17 ± 0.06	**< 0.05**	
	**Darkness (n = 7)**	5.64 ± 0.09	5.65 ± 0.09	5.91 ± 0.09	5.93 ± 0.08	> 0.05	
**Retinal thickness (mm)**	**Control (n = 5)**	0.23 ± 0.01	0.23 ± 0.01	0.24 ± 0.00	0.24 ± 0.00	> 0.05	> 0.05
	**Unresticted vision (n = 7)**	0.24 ± 0.00	0.25 ± 0.00	0.24 ± 0.01	0.24 ± 0.00	> 0.05	
	**Darkness (n = 7)**	0.23 ± 0.00	0.24 ± 0.00	0.23 ± 0.01	0.23 ± 0.01	> 0.05	
**Choroidal thickness (mm)**	**Control (n = 5)**	0.11 ± 0.01	0.11 ± 0.01	0.13 ± 0.00	0.12 ± 0.00	> 0.05	**< 0.05**
	**Unresticted vision (n = 7)**	0.12 ± 0.01	0.13 ± 0.01	0.14 ± 0.01	0.13 ± 0.00	**< 0.01**	
	**Darkness (n = 7)**	0.12 ± 0.01	0.12 ± 0.01	0.13 ± 0.01	0.13 ± 0.01	> 0.05	
**Axial length (mm)**	**Control (n = 5)**	9.46 ± 0.04	9.45 ± 0.04	9.66 ± 0.03	9.71 ± 0.04	> 0.05	> 0.05
	**Unresticted vision (n = 7)**	9.46 ± 0.06	9.45 ± 0.06	9.72 ± 0.06	9.80 ± 0.06	**< 0.05**	
	**Darkness (n = 7)**	9.24 ± 0.09	9.23 ± 0.10	9.54 ± 0.09	9.58 ± 0.07	> 0.05	
**Refractive error (D)**	**Control (n = 4)[a]**	-1.23 ± 0.25	-1.02 ± 0.36	0.19 ± 0.44	-0.64 ± 0.36	> 0.05	> 0.05
	**Unresticted vision (n = 6)[b]**	-0.62 ± 0.34	-0.29 ± 0.27	-0.48 ± 0.25	-2.02 ± 0.59	**< 0.05**	
	**Darkness (n = 7)**	0.20 ± 0.54	-0.70 ± 0.86	1.05 ± 0.70	-0.27 ± 0.74	> 0.05	

Control: Continuous lens wear without interruptions

Unrestricted vision: Lens wear interrupted with normal vision while the animals were kept in the drum

Darkness: Lens wear interrupted with darkness

p (t-test): Change in the experimental and fellow eyes was compared using paired, 2-tailed *Student*'s t-test

p (ANOVA): The relative change in 3 groups was compared using ANOVA

p values with statistical significance are shown in bold and unlined

a: Refractive error was measured in 4 out of the 5 animals

b: Refractive error was measured in 6 out of the 7 animals
