# Supplementary material for: Temporal properties of positive and negative defocus on emmetropization
Source: Sci Rep. 2022 Mar 4;12:3582. doi: 10.1038/s41598-022-07621-6 (PMC8897502; doi:10.1038/s41598-022-07621-6)
Supplement: Supplementary file 2 — Supplementary Table S2. [file 41598_2022_7621_MOESM2_ESM.pdf]

Supplemental Table S2. Ocular biometry and refractive state for the experimental (X, OD) and control eyes (N, OS) at baseline and after 4 weeks of -5D lens treatment. Data are shown as mean ± SEM.

	**Treatment**	**Baseline**		**4 weeks later**		**p**	**p**
		X (OD)	N (OS)	X (OD)	N (OS)	**(t-test)**	**(ANOVA)**
**Anterior chamber depth (mm)**	**Control (n = 13)**	1.50 ± 0.02	1.50 ± 0.02	1.59 ± 0.02	1.60 ± 0.02	> 0.05	
	**Unrestricted vision (n = 8)**	1.51 ± 0.03	1.51 ± 0.03	1.61 ± 0.03	1.59 ± 0.03	> 0.05	> 0.05
	**Darkness (n = 6)**	1.46 ± 0.04	1.46 ± 0.02	1.54 ± 0.03	1.54 ± 0.03	> 0.05	
**Lens thickness (mm)**	**Control (n = 13)**	2.06 ± 0.02	2.05 ± 0.02	2.03 ± 0.02	2.02 ± 0.02	> 0.05	
	**Unrestricted vision (n = 8)**	2.15 ± 0.04	2.15 ± 0.04	2.07 ± 0.03	2.07 ± 0.03	> 0.05	> 0.05
	**Darkness (n = 6)**	2.13 ± 0.04	2.13 ± 0.04	2.11 ± 0.03	2.10 ± 0.03	> 0.05	
**Vitreous chamber depth (mm)**	**Control (n = 13)**	5.86 ± 0.10	5.86 ± 0.10	6.22 ± 0.08	6.10 ± 0.08	**< 0.001**	
	**Unrestricted vision (n = 8)**	5.76 ± 0.04	5.74 ± 0.05	6.08 ± 0.05	6.05 ± 0.04	> 0.05	> 0.05
	**Darkness (n = 6)**	5.65 ± 0.05	5.67 ± 0.06	5.97 ± 0.06	5.92 ± 0.06	> 0.05	
**Retinal thickness (mm)**	**Control (n = 13)**	0.23 ± 0.00	0.23 ± 0.00	0.22 ± 0.01	0.23 ± 0.00	**< 0.05**	
	**Unrestricted vision (n = 8)**	0.22 ± 0.01	0.22 ± 0.01	0.25 ± 0.00	0.23 ± 0.01	> 0.05	**< 0.05**
	**Darkness (n = 6)**	0.24 ± 0.00	0.23 ± 0.01	0.23 ± 0.01	0.24 ± 0.00	> 0.05	
**Choroidal thickness (mm)**	**Control (n = 13)**	0.12 ± 0.01	0.12 ± 0.01	0.11 ± 0.00	0.11 ± 0.01	> 0.05	
	**Unrestricted vision (n = 8)**	0.12 ± 0.00	0.12 ± 0.01	0.12 ± 0.00	0.11 ± 0.01	> 0.05	> 0.05
	**Darkness (n = 6)**	0.14 ± 0.01	0.13 ± 0.01	0.13 ± 0.00	0.14 ± 0.01	**< 0.05**	
**Axial length (mm)**	**Control (n = 13)**	9.42 ± 0.11	9.41 ± 0.10	9.84 ± 0.08	9.72 ± 0.08	**< 0.01**	
	**Unrestricted vision (n = 8)**	9.42 ± 0.04	9.41 ± 0.03	9.76 ± 0.04	9.71 ± 0.03	> 0.05	> 0.05
	**Darkness (n = 6)**	9.24 ± 0.05	9.26 ± 0.04	9.62 ± 0.04	9.55 ± 0.05	> 0.05	
**Refractive error (D)**	**Control (n = 6)[a]**	-0.24 ± 0.39	-0.33 ± 0.48	-3.18 ± 0.61	-1.25 ± 0.33	**< 0.01**	
	**Unrestricted vision (n = 8)**	-0.64 ± 0.68	-1.11 ± 0.76	-2.10 ± 0.62	-1.64 ± 0.51	> 0.05	> 0.05
	**Darkness (n = 6)**	-0.71 ± 0.60	-0.89 ± 0.40	-2.02 ± 0.34	-1.07 ± 0.59	> 0.05	

Control: Continuous lens wear without interruptions

Unrestricted vision: Lens wear interrupted with normal vision while the animals were kept in the drum

Darkness: Lens wear interrupted with darkness

p (t-test): Change in the experimental and fellow eyes was compared using paired, 2-tailed *Student*'s t-test

p (ANOVA): The relative change in 3 groups was compared using ANOVA

p values with statistical significance are shown in bold and unlined

a: Refractive error was measured in 6 out of the 13 animals
